# Effects of proactive control and reward incentives on the inhibition of sequentially linked motor actions

**DOI:** 10.1007/s00426-026-02407-1

**Published:** 2026-10-21

**Authors:** Chris M. Dodds, Lidia Falcon-Hernandez, Jessica Wilson

**Affiliations:** Department of Psychology, University of Exeter, Washington Singer Building, Exeter, EX4 4QG UK

## Abstract

Effects of proactive control on response inhibition have previously been studied using tasks such as the stop signal task in which participants are required to inhibit a single, isolated motor response. However, skilled, coordinated behavior often involves the execution of learned sequences of motor actions in which each action triggers the next with a degree of automaticity. Across three experiments, we investigated the effects of proactive control and reward incentives on the frequency of errors in a motor sequence learning task that requires inhibition of sequentially triggered motor actions. We observed graded effects of proactive control on inhibition success. Although cues indicating an upcoming stop trial led to a marked reduction in the frequency of slips-of-action, errors continued to occur despite significant cue-induced proactive slowing. When cues were combined with incentives for successful stopping, proactive control increased further, with an additional reduction in frequency of slips-of-action. These findings show that the effectiveness of proactive control in suppressing automatically triggered actions within learned sequences is jointly determined by the depth of proactive slowing and the motivational context.

## Introduction

Automatic routines – learned sequences of consecutively triggered actions – form the backbone of a wide variety of human behaviors, from simple habits (Graybiel, 1998; Balleine & Dezfouli, 2019) through to complex skills (Viviani & Laissard, 1996; Shinar et al., 1998). Over time, individual actions repeatedly performed together in the same order can form linked sequences in which the initiation of the initial action in the sequence leads to automatic execution of the entire chain of actions (Graybiel & Grafton, 2015). Such action ‘chunking’ enables efficient performance of a wide range of everyday routine activities, with little requirement for the involvement of resource-intensive cognitive control processes (Dezfouli & Balleine, 2012; Sakai et al., 2003; Savalia et al., 2016; Smith & Graybiel, 2016).

However, automatic execution of action sequences can be problematic when contextual changes demand a more flexible response. Failure to activate cognitive control processes involved in the inhibition of motor actions can result in common everyday errors – so-called ‘slips-of-action’ (Reason, 1984, 2009). Whilst there are many different varieties of action slips, some errors clearly result from the automatic triggering of an action that is embedded within a well-learned sequence. Numerous examples can be found in everyday domestic contexts. For example, making a cup of tea or coffee can be represented in a hierarchical manner, with the overarching goal (make tea/coffee) consisting of a subordinate sequence of motor actions (fill the kettle, turn the kettle on, pour the boiled water over the tea/coffee, add milk). Lapses of attention can reveal the automatic nature of action triggering within such sequences, for example when one accidentally flicks the switch to turn the kettle on again after having already poured the boiling water into the cup - whilst contextual changes can also lead to errors, for example when one adds milk to a coffee that a friend requested black. Musicians will also be familiar with these kinds of errors – learning a piece of music can sometimes involve changing the final note or cluster of notes at the end of a repeating passage, requiring inhibition of the default action or relearning a particular sequence that one has initially learnt to play in a suboptimal fashion. In all of these examples, the subjective sense is often that ongoing behavior is biased towards the default sequence, and that a substantial amount of control is required to overcome the well-rehearsed routine.

Cognitive control refers to the collection of executive operations that are recruited when automatic behavioral routines cannot cope with novel or challenging task demands (Miller & Cohen, 2001). The dual mechanisms of control theory (Braver, 2012) states that cognitive control operations can be divided into proactive and reactive mechanisms. Within this framework, reactive control is conceptualised as a process of responding to a rapid change in the environment that demands an immediate change or cancellation of ongoing behaviour – for example, inhibiting the action of opening the car door when you notice a cyclist appearing in the rear-view mirror. Proactive control, on the other hand, is conceptualised as a slower, more careful process involving the active maintenance of goal representations in anticipation of an upcoming need for control – for example, opening the car door slowly and carefully while scanning for approaching cyclists and preparing to cancel the action because you are on a busy road with lots of cyclists.

Response inhibition – the process of cancelling a prepotent or automatic motor action – is a key component of the machinery of cognitive control (Miyake et al., 2000) and is a crucial process for the successful avoidance of slips-of-action (Norman, 1981). An important question, therefore, concerns the extent to which proactive control can enhance inhibitory control. Studies that have examined the effects of proactive control on response inhibition have generally used the Stop Signal Task (SST) to measure inhibitory control (Doekemeijer et al., 2023; Leunissen et al., 2016; Messel et al., 2019, 2021; Ramautar et al., 2004; Verbruggen & Logan, 2009). The SST requires participants to respond to a ‘go stimulus’ on each trial, such as the direction of an arrow, and on occasional stop trials to withhold the go response when a ‘stop signal’ occurs shortly after the go stimulus. The most consistent finding from such studies is that manipulations of proactive control – for example, explicitly cueing participants about the relevance of stop signals, or varying the relative proportion of go and stop trials - lead to an increase in go trial RT, indicating a shift in strategy towards the prioritisation of slow, careful responding over response speed. Proactive control has also been found to significantly reduce stop signal reaction time (SSRT; Doekemeijer et al., 2023), which indexes the efficiency of stopping.

However, whilst the SST is a sensitive and well-validated test of response inhibition, it constitutes a rather narrow model of the inhibitory control of isolated motor actions. As outlined above, real life actions do not always occur in isolation but are often embedded within linked sequences of consecutively triggered actions, with the difficulty of inhibiting any particular action within such a sequence presumably dependent on the degree of automaticity of such sequential triggering. Logan (1982) previously investigated participants’ ability to inhibit actions embedded in sequences in the context of skilled typing, finding that participants were generally able to inhibit individual keystrokes when a stop signal was presented. However, as far as we are aware, no study has previously investigated how proactive control affects the ability to stop actions that form part of learned motor sequences. This is an important question not only for understanding the mechanisms underlying everyday flexible behavior, but also potentially for furthering our understanding of clinical conditions such as Obsessive Compulsive Disorder (OCD) in which compulsive repetitive behaviors have been hypothesised to involve a breakdown in the neurocognitive circuitry controlling the balance between goal-directed and habitual behavior (Gillan et al., 2011; Graybiel & Rauch, 2000). Indeed, progress in understanding such disorders may depend on the development of effective methods for studying the flexible control of sequence-based, habit-like behaviors (Robbins & Costa, 2017).

The aim of the present study, therefore, was to examine more specifically the effects of proactive control on motor response inhibition during visuomotor sequence learning. We recently developed a novel task that measures action switching during explicit visuomotor sequence learning (Dodds, 2025). Participants learned to make repeated sequences of keypresses to visual stimuli appearing in a repeated pattern and were occasionally required to switch to a novel action when one of the stimuli in the sequence appeared out of its usual position. Cueing participants about an upcoming switch trial led to engagement of proactive control (slower responding on the steps of the sequence preceding the switch) and a significant reduction in switch errors, suggesting that proactive control can modulate action switching performance. However, effects of proactive control were imperfect – errors continued to occur despite a significant level of proactive slowing, suggesting that the automaticity of actions in motor sequences may confer some resistance on those actions to the effects of proactive control.

An important unanswered question from the Dodds (2025) study is whether the effects of proactive control on switch errors were driven by effects on response inhibition or action selection. Studies using stop-change paradigms that require participants to replace a motor response with a different one suggest that action switching involves two separate processes operating in serial – a stop process followed by an action selection process (Camalier et al., 2007; Verbruggen et al., 2008). Similarly, in the action switching task employed in Dodds (2025) the process of switching to the novel action presumably required participants to both inhibit the default action and to select the new action. Thus, it is unclear whether the beneficial effects of proactive control in that study were driven specifically by an enhancement of the response inhibition process. Moreover, it is difficult to know whether the observation that proactive control was only partially successful at preventing errors was due to the high level of automaticity of the actions, and consequently the difficulty of inhibiting them, or was in fact due to the requirement to engage multiple control processes at the point of switching.

The task used in the experiments described here is based on the one employed in Dodds (2025), in that on each trial participants made six consecutive keypress responses to six consecutively presented blue squares that appeared in a repeating order in horizontally arrayed boxes on a screen. The key difference from that study was that instead of occasional changes to the sequence requiring an action switch, on a subset of trials one of the squares was red instead of blue and participants were required to withhold their response on that step of the sequence before continuing to respond as usual for the rest of the trial. Thus, the task in the present study was designed to directly test inhibitory control of actions that are automatically triggered as part of a well-learned visuomotor sequence. In all three experiments, the primary measure of stopping success was the frequency of ‘habit errors’ - stop trials on which the participant erroneously pressed the default response key, reflecting the expression of a previously learned habitual response rather than a deliberate action. The primary measure of proactive control was the degree to which, following a cue at the start of the trial indicating that the present trial would involve a stop, participants slowed down their responses on the steps of the sequence preceding the actual stop (‘pre-stop steps’) relative to RTs on go trials.

In experiment 1, we examined the extent to which cueing participants in advance of a stop trial would improve their ability to withhold the default response. The prediction was that such cueing would lead to participants engaging proactive control, evidenced by slower RTs on pre-stop steps relative to go trials, and that this would be accompanied by a reduction in the frequency of habit errors in cued relative to uncued blocks.

In experiment 2, all stop trials were pre-cued with the aim of maximising power to test more specific predictions about the link between proactive control and improved stopping performance, and to establish that performance of the sequence increases in automaticity over time by examining changes in patterns of responding across the steps of the sequence and across the duration of the task. In line with the results from our previous study on response switching during motor sequences (Dodds, 2025), we predicted that (a) proactive control would exert a graded effect on stopping, with significant proactive slowing preceding habit errors, but significantly greater proactive slowing preceding correct stop trials (b) go RTs would decrease, and frequency of habit errors would increase, across the six blocks of the task, indicating an increase in automaticity with learning, and (c) frequency of habit errors would increase when the stop occurred on steps 2 to 6 of the sequence relative to step 1, due to automatic triggering of the current action by the previous one.

Experiment 3 aimed to investigate whether limitations in the effects of proactive control on stopping performance suggested by the results of experiments 1 and 2 are fixed and immutable, or whether they may be at least in part accounted for by motivational factors. To examine this, we introduced a small monetary incentive for successful stopping and manipulated whether or not this reward was available across blocks. We predicted that (a) participants would show significantly greater cue-induced slowing on pre-stop steps in reward blocks relative to non-reward blocks, indicating a further reward-related increase in proactive control over and above that induced purely by cueing, and (b) a reduction in frequency of habit errors on cued stop trials in reward blocks relative to non-reward blocks, indicating a further reward-related increase in stopping success over and above that induced purely by cueing.

## Experiment 1

In experiment 1, we first aimed to establish whether cueing participants about an upcoming stop trial would induce proactive slowing, and whether this would lead to a reduction in habit errors. Participants performed the sequence task in 6 blocks, with 3 cued blocks and 3 uncued blocks in an interleaved order. Throughout this paper, we refer to a ‘trial’ as a whole sequence of 6 stimuli and responses, and a ‘step’ as one specific stimulus and response within a trial. Thus, a ‘go trial’ is a sequence of 6 steps, all of which required a go response; a ‘stop trial’ is a sequence of 6 steps, one of which required a withheld response; a ‘stop step’ is the specific step of a stop trial on which the square was red and the participant was required to withhold their response; and a ‘pre-stop step’ refers to all of the steps preceding the stop step. Finally, a ‘habit error’ is an incorrect response on a stop step of a stop trial on which the participant responded with the default response at that step of the sequence instead of withholding their response. We predicted, firstly, that participants would show significantly greater RT slowing on pre-stop steps in the cued condition relative to the uncued condition, and secondly that participants would make fewer habit errors in the cued condition relative to the uncued condition.

### Method

#### Participants

A pilot study was carried out to determine the sample size required to detect significant effects of cueing on proactive slowing and on frequency of habit errors. Ten participants were recruited from Prolific and completed the cued/uncued sequence task. Analysis revealed effect sizes of d = 0.53 and d = 1.76 for paired samples t-tests comparing cued vs. uncued bocks for RT and error frequency, respectively, and so the main study was powered to detect the smaller of the two effects. A G-power analysis revealed that a sample size of 30 would be required to detect an effect size of 0.53 for a paired samples t-test (2-tailed) with an alpha level of 0.05 and 80% power. For the main study therefore, 40 participants (19 females) were recruited from Prolific, with a mean age of 43.4 years (SD = 11.1). Thirty-eight participants were right-handed. Participants were paid £3.75 for taking part. Data were collected in 2025.

#### Task design and procedure

**Fig. 1:**
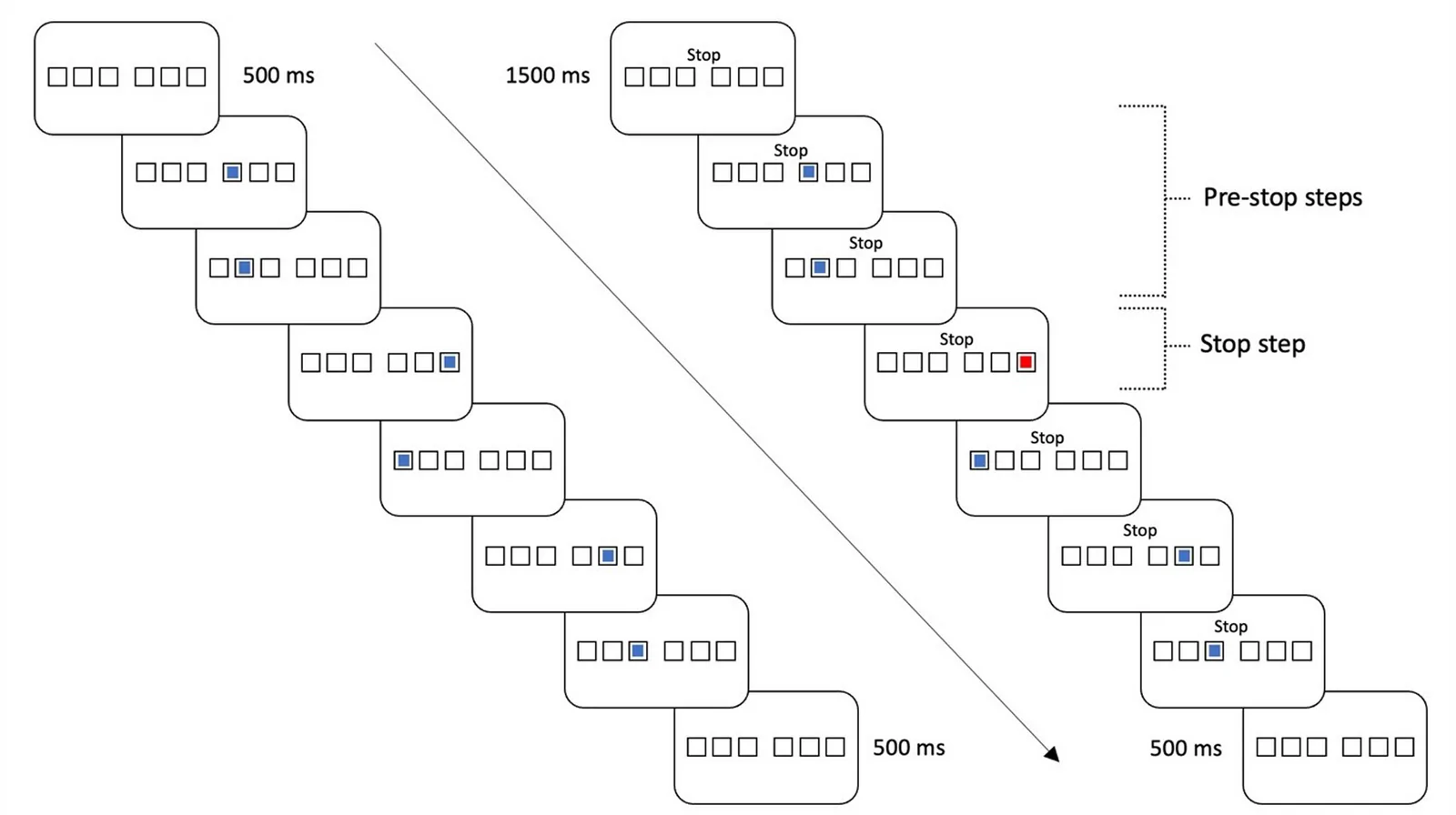
Sequence of events in a go trial (left) and a cued stop trial (right) in experiment 1. Participants placed their fingers on the A, S, D, j, K and L keys of the keyboard and on each step of the trial pressed the key corresponding to the location of the blue square, withholding the response if a red square appeared (step 3 of the sequence in this figure)

The task was programmed and run using the online experiment software Gorilla (www.gorilla.sc). The task required participants to respond to the locations of blue squares that appeared within boxes arranged horizontally on the screen (see Fig. 1). The task was composed of go trials and stop trials. On each go trial, six blue squares appeared, one at a time, in different boxes in a repeating, predictable sequence. The sequence was randomly determined at the start of the experiment and was different for each participant. For each participant the sequence remained the same for the whole experiment. Throughout this paper, we refer to a ‘trial’ as the entire sequence of 6 blue squares, and a ‘step’ as a single presentation of a single blue square.

On stop trials, the sequence was the same as on go trials except that one of the squares was red instead of blue and participants were required to withhold their response on this step (the ‘stop step’). Each of the steps on a stop trial that precedes the stop step is called a ‘pre-stop step’. For example, on a stop trial in which the stop step occurs on step 4 of the sequence, step four is the stop step, and steps one to three are the pre-stop steps.

There were six blocks of 54 trials, with 48 go trials and six stop trials in each block, producing a total of 288 go trials and 36 stop trials across the whole task, and a stop to go trial ratio of 1:8. On a stop trial, the stop step could occur at any of the six steps in the sequence. Within each block, there was one stop trial with a stop at each step of the sequence. The order of all trials within a block was randomised, with the limitation that there were never two contiguous stop trials. For each participant there were three blocks with cued stop trials and three blocks with uncued stop trials. Cued/uncued blocks were interleaved, with half of the participants experiencing a cued block first (ABABAB order) and the other half of the participants experiencing an uncued block first (BABABA order).

The task began with an instruction screen asking participants to place the ring, middle and index fingers of their left hand on the A, S and D keys, and the index, middle and ring fingers of their right hand on the J, K and L keys, and to keep them there. A picture of a hand accompanied this instruction, with the fingers pointing at the appropriate keys. Participants were then told that they would be required on each trial to press the key corresponding to the location of each blue square as quickly and accurately as possible. This instruction screen was followed by four practice go trials. After the practice trials a further instruction screen explained that on some trials one of the squares would be red instead of blue, and that they should not press for that square, but wait for the next blue square to appear and continue to press as normal. After another set of three practice trials, including two uncued stop trials, another set of instructions appeared explaining that some stop trials would be cued, with the word ‘stop’ above the boxes at the start of the trial, and others would be uncued, and that the task remained exactly the same on these trials – press for blue squares, don’t press for red squares. There followed three more practice trials including two cued stop trials before the main task began.

Figure 1 shows the sequence of events on a go trial (left) and a cued stop trial (right). On go trials and uncued stop trials, at the start of the trial six empty squares appeared on the screen arranged horizontally across the centre of the screen. The empty squares remained visible for 1500 ms, after which six blue squares appeared consecutively. Each blue square remained visible until the participant responded by pressing the appropriate key on the keyboard, at which point the current blue square disappeared and the next one appeared. At the end of the sequence the empty boxes were again presented for 500 ms.

On a cued stop trial, the six empty squares appeared on the screen together with the word ‘Stop’ in the centre of the screen just above the squares. The empty squares together with the cue remained visible for 1500 ms before the first blue square appeared. The cue remained visible throughout the rest of the trial. At the end of the sequence the empty boxes were again displayed for 500 ms. At the end of each block, participants were given 30 s to rest before the next block commenced with a visible countdown from 5 to 1.

### Transparency and openness statement

Data were analyzed using R, version 4.4.1 (R Core Team, 2025) and the package ggplot, version 3.5.1 (Wickham, 2016). All data and analysis code are available at https://figshare.com/s/a25a54d4e57672468a7c. We report how we determined the sample size, all data exclusions (if any), all manipulations, and all measures in the study. This study’s design and its analysis were not preregistered.

### Compliance with ethical standards

The experiment was reviewed and approved by the Exeter Psychology Research Ethics Committee (application ID 6742165). The authors have no relevant financial or non-financial interests to disclose.

### Data processing and analysis

Across all three experiments, any participant with go step accuracy < 85% was excluded from all data analyses. The rationale for this approach was that if a participant consistently responded incorrectly on at least one step of every go trial, this would suggest a lack of compliance or motivation to engage properly with task instructions, and their go step accuracy would be 83.3%. Analysis of accuracy data for all three experiments focused on the frequency of ‘habit errors’ – frequency of stop steps in which the participant failed to withhold their response and pressed the default key for that step of the sequence that they would have pressed on a go trial. Analysis of proactive slowing focused on RTs on the steps of a stop trial preceding the stop step (‘pre-stop steps’). For each participant, median pre-stop step RT was calculated by taking the median RT across all pre-stop steps on all stop trials. Across all three experiments, figures display standard error bars which represent within-subject SEMs calculated using the Morey (2008) method.

### Results

Mean accuracy for go steps across all participants in experiment 1 was 98% correct (SD = 1.8). The lowest level of go step accuracy for any participant was 93%. Therefore, in experiment 1 no participants were excluded from data analyses. As a manipulation check, a one-sample t-test confirmed that habit error rates were significantly greater than zero (M = 19.6%, SD = 12.0%), t(39) = 14.24, *p* < .001 (test conducted on sqrt-transformed values), demonstrating that the task successfully induced habitual responding across the sample.

**Fig. 2:**
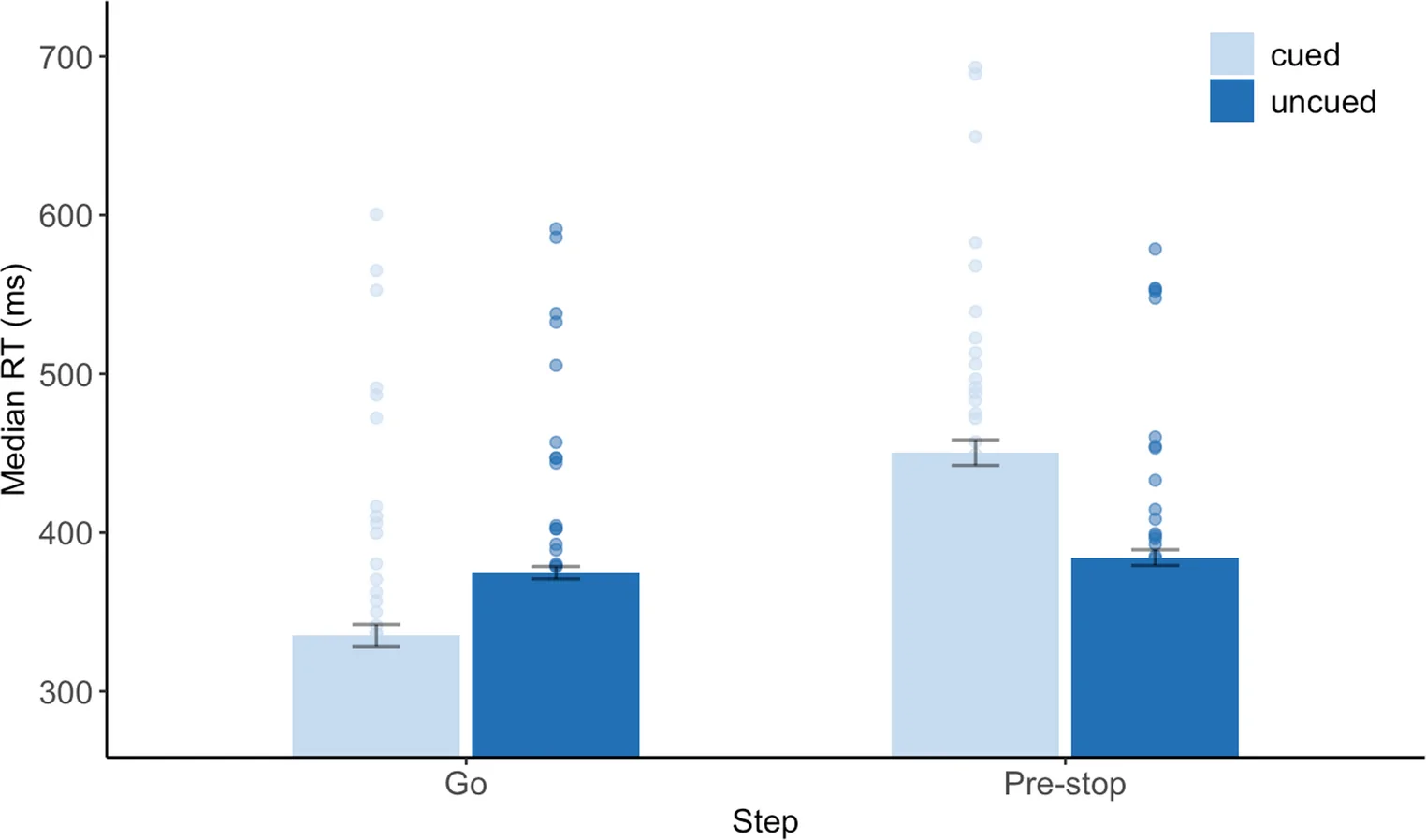
Means of median RTs on go steps and pre-stop steps in cued and uncued blocks in experiment 1. Error bars represent SEM; individual points represent participant-level median RTs

A paired samples t-test on square root transformed percentage errors on stop steps revealed, as predicted, significantly higher frequency of errors in the uncued condition (M = 30.5%, SD = 18.1) than in the cued condition (M = 8.6%, SD = 11), t(39) = 7.8, *p* < .001, d = 1.45. To examine effects of cueing on proactive slowing, a repeated measures ANOVA was carried out with the factors cue condition (cued, uncued) and step (go, pre-stop) with median RTs as the dependent variable. This revealed significant main effects of cue condition, *F*(1, 39) = 4.66, *p* = .037, η² = 0.005, and step, *F*(1, 39) = 75.38, *p* < .001, η²_g_ = 0.106, and a significant cue condition x step interaction, *F*(1, 39) = 102.55, *p* < .001, η²_g_ = 0.078. Follow-up paired samples t-tests confirmed that median pre-stop RTs were significantly slower in the cued condition (M = 450 ms, SD = 91), than the uncued condition (M = 384 ms, SD = 83), t(39) = 7.3, *p* < .001, d = 0.75, whilst median go RTs were significantly faster in the cued condition (M = 335 ms, SD = 104), than the uncued condition (M = 375 ms, SD = 87), t(39) = 5.8, *p* < .001, d = 0.38 (see Fig. 2).

To examine whether the magnitude of cue-induced proactive slowing was associated with the magnitude of cue-induced reduction in inhibitory errors, we conducted a participant-level correlation between the two experimental effects. For each participant, proactive slowing was calculated as the difference in median pre-stop RT between the cued and uncued conditions, with larger positive values indicating greater cue-induced slowing. The corresponding reduction in habit errors was calculated as the difference between square-root-transformed percentage errors in the uncued and cued conditions, with larger positive values indicating a greater reduction in errors following cueing. In the full sample, there was a significant positive correlation between RT slowing and error reduction, *r*(38) = 0.43, *p* = .006, 95% CI [0.13, 0.65]. Given the presence of one participant with an unusually large RT effect (314 ms), we repeated the analysis excluding this participant. The correlation remained significant, *r*(37) = 0.36, *p* = .024, 95% CI [0.05, 0.61], indicating that the observed association was not dependent on this outlier.

### Discussion

The results of experiment 1 showed, as predicted, that cueing participants about an upcoming stop trial led to engagement of proactive slowing (on the steps of the stop trial preceding the stop) and a significant reduction in the frequency of habit errors. These data therefore demonstrate clearly that engagement of proactive control is an effective mechanism for reducing inhibitory control errors in contexts where the action to be inhibited is embedded in a learned sequence of motor actions. Furthermore, we examined whether individual differences in the magnitude of cue-induced proactive slowing were associated with the magnitude of the reduction in habit errors. There was a positive association between these effects, such that participants who showed greater cue-induced slowing also tended to show a greater reduction in habit errors. This provides further evidence for a relationship between the engagement of proactive control and the reduction of inhibitory control errors.

## Experiment 2

In experiment 1, although cueing led to a large and significant improvement in the accuracy of inhibitory control, participants continued to make habit errors on around 8% of cued stop trials. A key question, therefore, is why do participants fail to inhibit a response when they can be 100% confident that a stop is going to occur on the present trial? One possibility is that on some trials participants simply fail to pay attention to the cue, leading to a failure to engage proactive control and a subsequent failure to inhibit the key response. This is plausible - lapses of attention occur frequently in everyday life (Norman, 1981; Reason, 1984, 2009) and are regularly cited as an explanation for the frequent commission errors that occur in simple tests of sustained attention such as the Sustained Attention to Response Task (Manly et al., 1999). However, an alternative possibility is that participants do in fact engage proactive control on habit error trials, but to a lesser extent than they do on correct stop trials. If this is the case, it would suggest that participants have paid attention to the cue, but that the level of proactive slowing is insufficient to exert control over the default action at that point in the sequence due to the high level of automatic triggering by the previous action in the sequence.

On the basis of the results of experiment 1, it is not possible to distinguish between the different explanations outlined above due to the low numbers of habit errors in the cued condition – participants made an average of approximately 1.5 habit errors in the cued condition. Therefore, in experiment 2 participants performed six blocks of cued stop trials in order to maximise the number of cued stop trials and enable a statistical comparison of average RTs preceding habit errors and correct stop responses. This design follows that adopted in experiment 2 of Dodds et al. (2025) which showed a graded effect of proactive control during action switching, whereby participants engaged proactive control on error trials but to a lesser extent than on correct switch trials. Here, we hypothesise that the pattern of graded proactive control in the previous study was due to the difficulty of inhibiting automatically triggered actions embedded in sequences, and therefore we predicted that a similar effect would be observed here – that pre-stop RTs on habit error trials would be intermediate between go RTs and pre-stop RTs on correctly executed stop trials.

Another key finding from the Dodds (2025) study was that action switching errors increased in frequency throughout the sequence, such that errors were more frequent when the switch occurred on later steps of the sequence than when it occurred on earlier steps. This pattern of results was interpreted as being due to errors being more likely when the default action is automatically triggered by the previous action in the sequence. This interpretation is consistent with the fact that the increase in errors was particularly steep between step 1 and step 2 (because step 2 is triggered by the previous step, but step 1 is not). However, it is also somewhat difficult to reconcile with the observation that errors continued to increase, albeit less dramatically, between steps 2 and 6 of the sequence. Indeed, an alternative explanation might be that participants attended to the cue at the start of the trial, leading to immediate prioritisation of the switch goal at step 1, but that attention to the goal of switching waned over the course of the trial.

Motivated by these considerations, a secondary aim of experiment 2 was to examine whether habit errors in the sequence stopping task are sensitive to similar sequential effects as those observed for action switching in the Dodds (2025) study, i.e. whether they increase across steps of the sequence, and whether such an increase conforms to an account in which the difficulty of inhibition is driven by either attentional or inhibitory demands. The attentional account predicts that habit errors should increase in a linear fashion across all six steps of the sequence. The inhibition account predicts that habit errors should increase between steps 1 and 2, but there should be no difference in frequency of errors between steps 2 and 6 (because sequential triggering by a prior action is equivalent at each of these steps).

Finally, an additional aim of experiment 2 was to investigate learning of the sequence over the course of the task, and the extent to which performance of the task increases in automaticity over this time course. If participants do learn the sequence, we should expect participants to get faster at responding to the default sequence over time. If performance of the sequence task increases in automaticity, we should expect to see an increase in the difficulty of inhibiting the default keypress on stop steps over time. We therefore predicted that (a) RTs on all steps of go trials would show a linear decrease and (b) habit errors would show a linear increase across the six blocks of the task.

### Method

#### Participants

A pilot study was carried out to determine the sample size required to detect a difference in the frequency of habit errors across steps of the sequence, and to detect proactive slowing on correct stop trials and habit errors. Twenty participants were recruited from Prolific and completed a version of the sequence stopping task in which all stop trials were cued (see details below). A linear mixed model revealed a very small coefficient for the linear effect of step on habit errors (b = 0.13). We therefore powered the study to detect the difference between the frequency of habit errors at step 1 and step 2. The pilot data revealed a Cohen’s d effect size of 0.45 for this effect, with a G-power analysis showing that a sample size of *N* = 41 would be required to detect an effect of this size with 80% power and alpha = 0.05 (2-tailed). Cohen’s d effect sizes for the comparisons of median pre-stop RTs on habit error trials and correct stop trials vs. go trials were 0.62 and 0.59 respectively, and so the study was adequately powered to detect significant proactive slowing on stop trials, whether those trials were responded to correctly or incorrectly. For the main experiment, 55 participants (24 females) were recruited from Prolific, with a mean age of 36 years (SD = 10.5). 48 participants were right-handed. Participants were paid £3.75 for taking part. The experiment was reviewed and approved by the Exeter Psychology Research Ethics Committee (application ID 6742165). Data were collected in 2025.

#### Task design and procedure

The task design and procedure were exactly the same as experiment 1 except that participants completed 6 blocks with cued stop trials (i.e. all stop trials were cued).

### Results

Data from four participants were excluded from all analyses due to go step accuracy < 84%. As a manipulation check, a one-sample t-test confirmed that habit error rates were significantly greater than zero (M = 14.5%, SD = 20.5%), t(50) = 10.66, *p* < .001 (test conducted on sqrt-transformed values), demonstrating that the task successfully induced habitual responding across the sample. Means of median RTs on go steps and pre-stop steps for each step of the sequence are shown in Table 1.

**Table 1:** Mean (SD) of median go and pre-stop RTs by step

Trial step	Go RT	Pre-stop RT
Step 1	379 (65)	474 (118)
Step 2	343 (68)	431 (112)
Step 3	341 (56)	444 (126)
Step 4	332 (76)	465 (108)
Step 5	325 (68)	498 (104)
Step 6	291 (55)	—

**Fig. 3:**
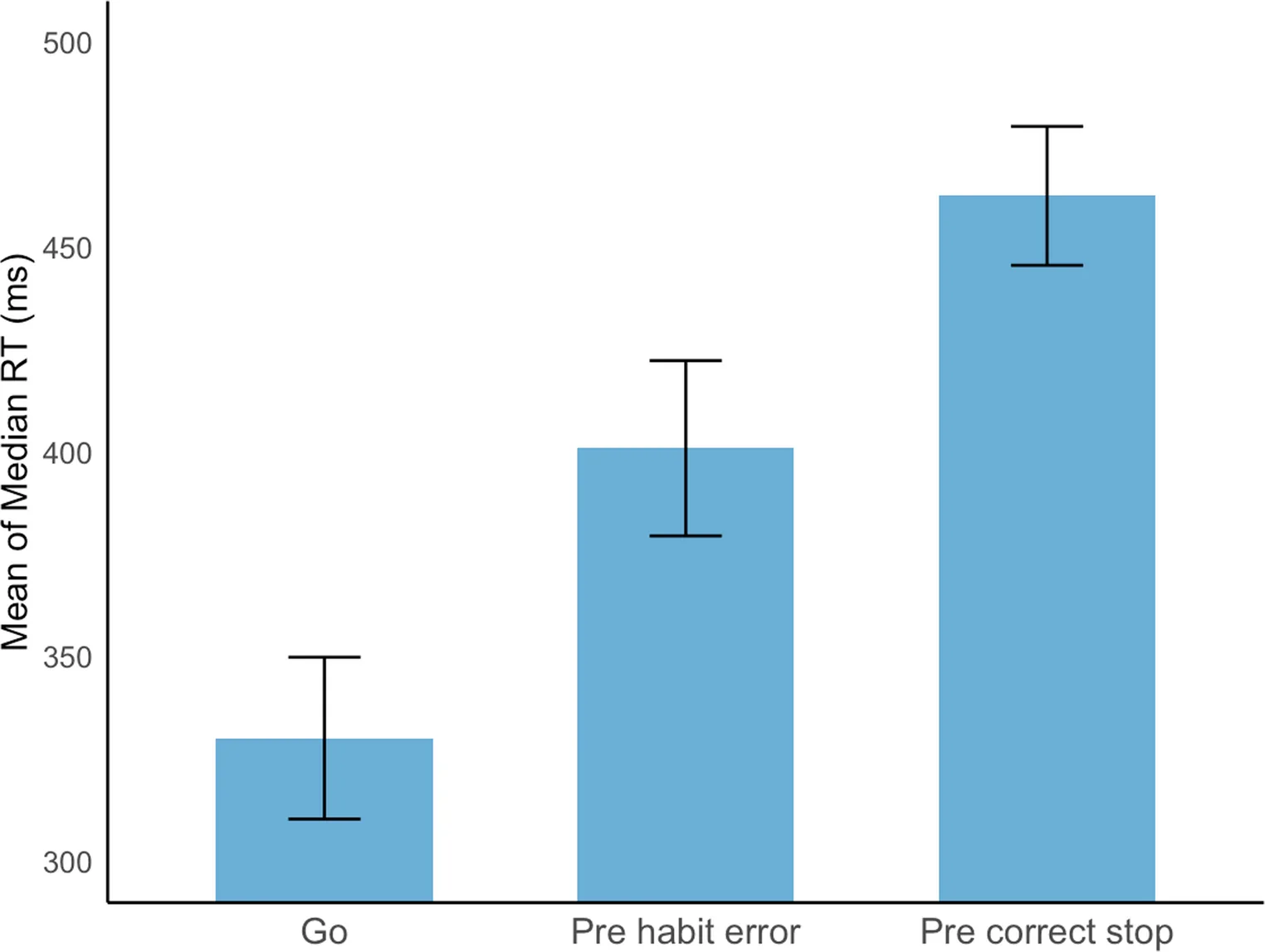
Means of median RTs on go steps, pre-stop steps on habit error trials, and pre-stop steps on correct stop trials in experiment 2. Error bars represent SEM

To examine whether RTs differed across go steps, habit errors and correct stops, a repeated measures ANOVA was carried out with the factor step (go, habit error, correct stop) with median RT as the dependent variable, including only data from participants with at least one trial of each step type (*N* = 43). Mauchly’s test indicated that the assumption of sphericity was not violated, W = 0.98, *p* = .63. There was a significant effect of step type on median RTs, F(2, 84) = 43.86, *p* < .001, n^2^_g_ = 0.16. Follow-up paired-samples t-tests confirmed that RTs were significantly slower on pre-stop steps of habit error trials (M = 401 ms, SD = 140) than on go steps (M = 330 ms, SD = 129), *t*(42) = 4.7, *p* < .001, *d* = 0.72. RTs were also significantly slower on pre-stop steps of correct stop trials (M = 462 ms, SD = 111) than on go steps, *t*(42) = 9.3, *p* < .001, *d* = 1.4. Finally, RTs on pre-stop steps of correct stop trials were significantly slower than those on pre-stop steps of habit error trials, *t*(42) = 4.7, *p* < .001, *d* = 0.72 (see Fig. 3).

**Fig. 4:**
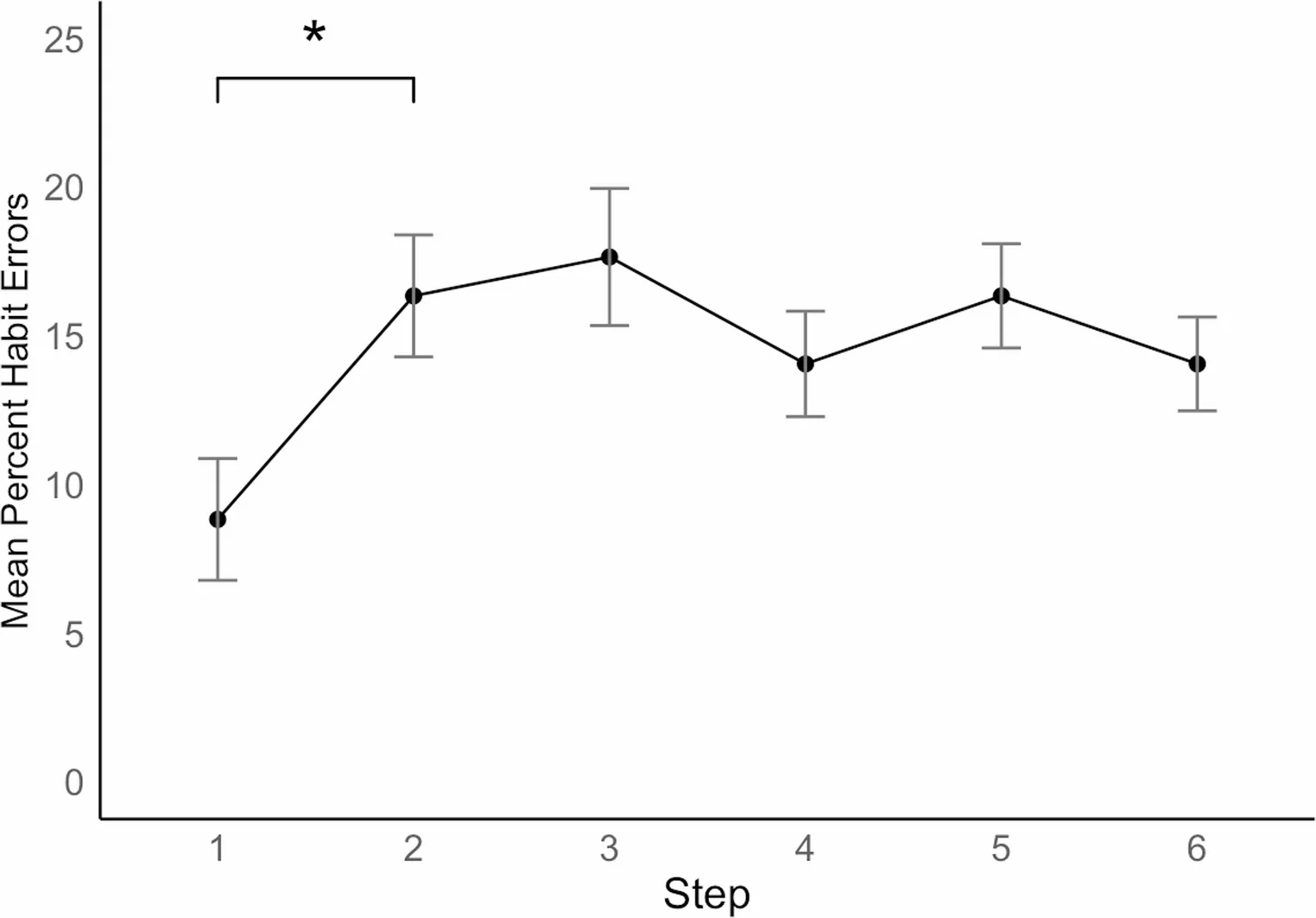
Mean percentage of habit errors when the stop step occurred at each step of the sequence on stop trials. * = significant difference at *p* < .05. Error bars represent SEM

To test whether frequency of habit errors increased in a linear fashion across the steps of the sequence, a linear mixed-effects model was fit with step (1 to 6) as the predictor and square root transformed percentage of habit errors as the dependent variable. The model indicated that the linear effect of step was not significant, b = 0.1, SE = 0.07, t(254) = 1.3, *p* = .2, suggesting no evidence for a linear increase or decrease in habit errors across steps. To examine the specific difference in frequency of errors between step 1 and step 2, a second model was fit with step entered as a categorical predictor. Relative to step 1, participants made significantly more habit errors at step 2, b = 1.02, SE = 0.43, t(250) = 2.37, *p* = .019, indicating that error rates were higher at step 2 than step 1 (see Fig. 4).

To complement the LMM analysis, and to quantify the evidence against any further increase in errors beyond step 2, we fitted a Bayesian linear mixed model (brms; Bürkner, 2017) to the square-root-transformed percentage of errors, with step (centred at step 2) as a fixed effect and by-participant random intercepts and slopes. We used weakly informative priors and confirmed convergence (all R̂ = 1.00, no divergent transitions). The posterior for the slope was centred near zero and slightly negative (*b* = − 0.07, 95% CrI − 0.28, 0.13; P(*b* > 0) = 0.23). The upper limit of the interval corresponds to an increase of < 3% points between steps 2 and 6, so the data exclude any appreciable linear increase in errors.

**Fig. 5:**
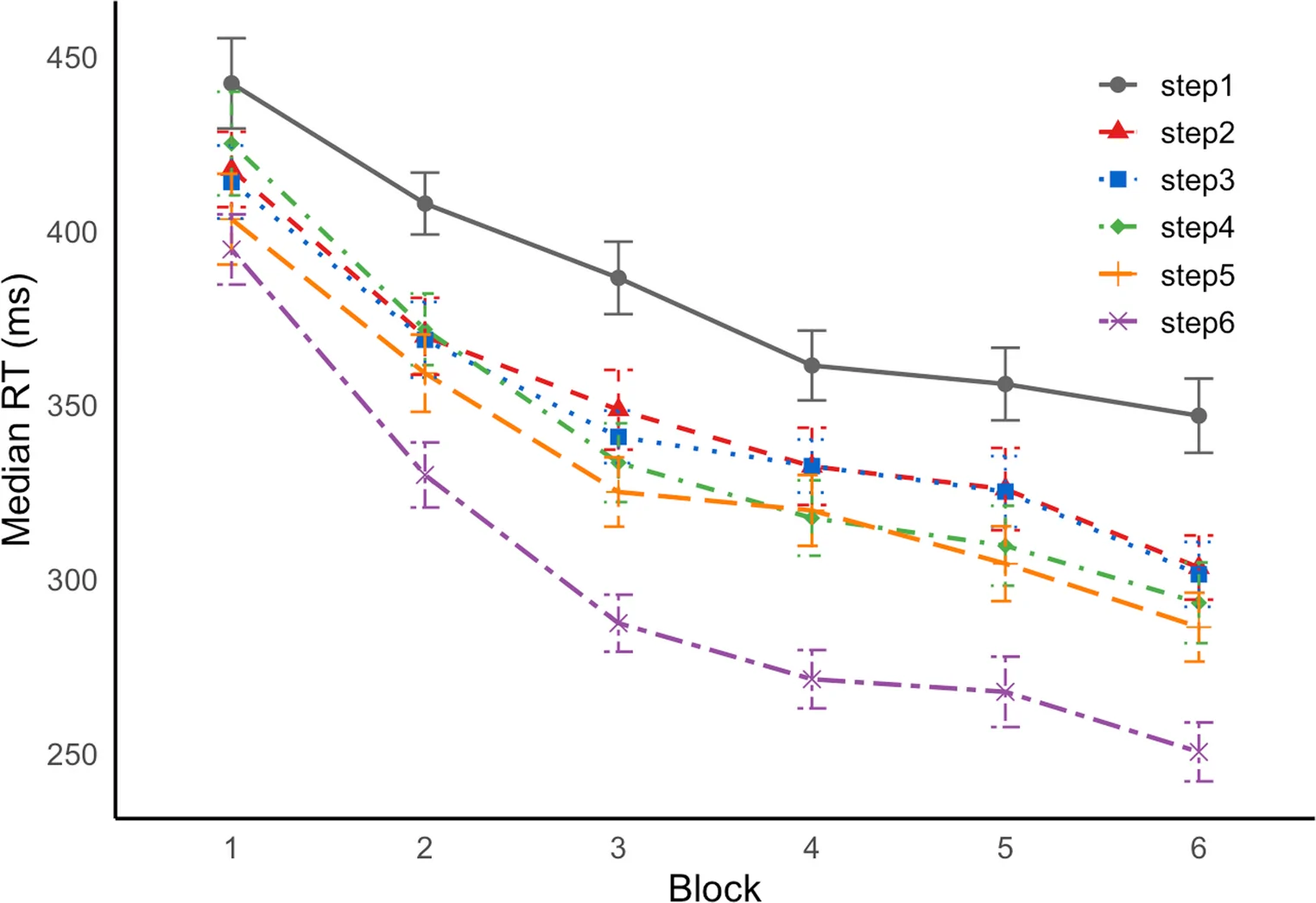
Means of median go trial RTs for each step of the sequence by block. Error bars represent within-subject SEMs

To test whether go trial RTs changed across the six blocks of the task, and whether this varied by step, a linear mixed-effects model was fitted to median go trial RTs, with block (1 to 6) and step (1 to 6) and their interaction entered as fixed effects, and random intercepts for participants. There was a significant main effect of block, *b* = − 17.44, *SE* = 2.37, *t*(1782) = − 7.36, *p* < .001, indicating faster responses across blocks. There was also a significant main effect of step, *b* = − 8.91, *SE* = 2.37, *t*(1782) = − 3.76, *p* < .001, such that RTs decreased across the steps of the sequence. The block x step interaction was also significant, *b* = − 1.31, *SE* = 0.61, *t*(1782) = − 2.14, *p* = .032, indicating that the effect of block varied across steps of the sequence. As you can see in Fig. 5, RTs to all steps of the sequence showed a linear decline across the six blocks, with a steeper decline later steps, and the most pronounced decline for step 6.

**Fig. 6:**
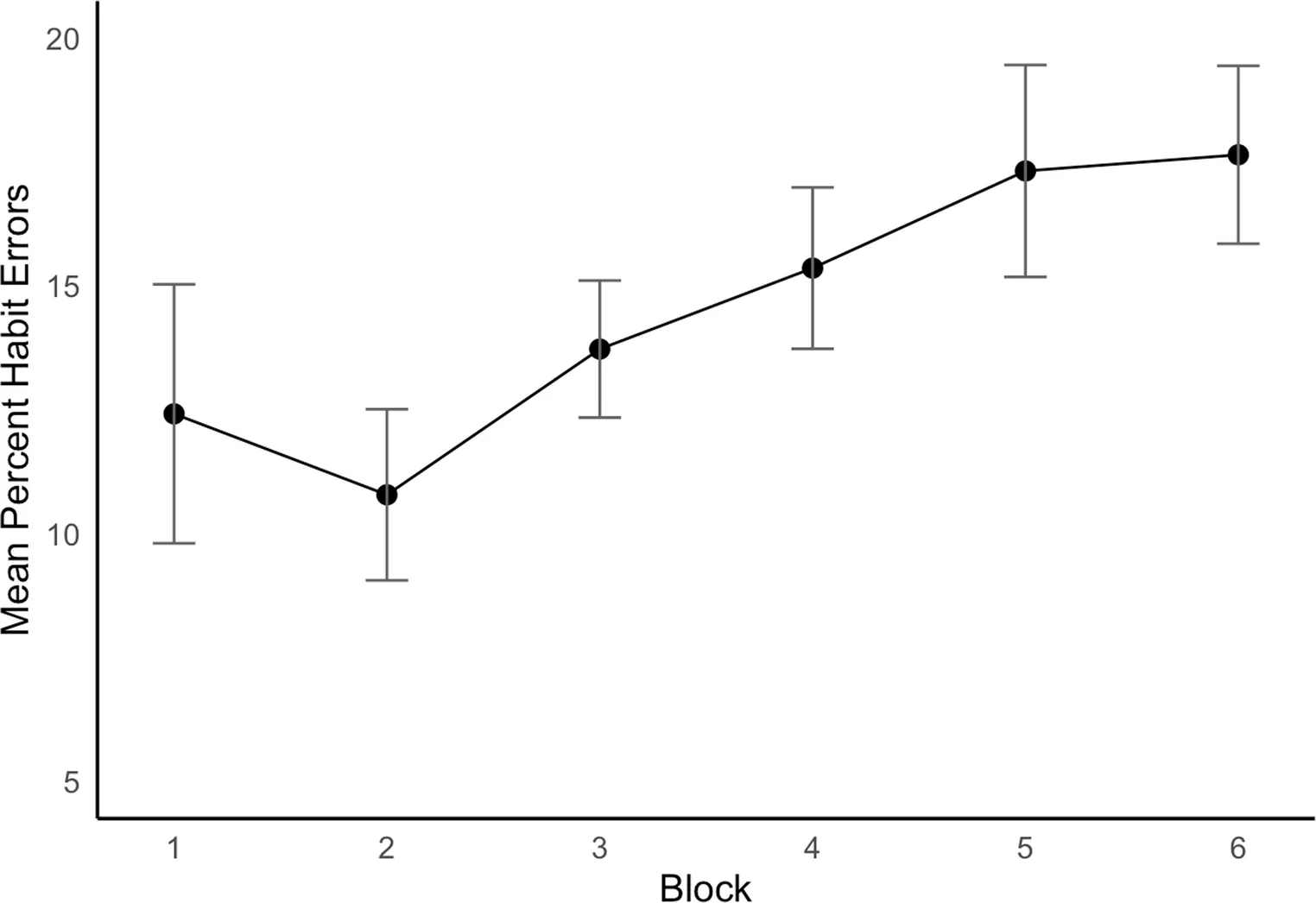
Mean percent habit errors by block. Error bars represent within-subject SEMs

To test whether frequency of habit errors changed across the six blocks of the task, a linear mixed-effects model was fitted to square root transformed percentage habit errors, with block (1 to 6) as fixed effects, and random intercepts for participants. The model showed a significant positive effect of block number on habit errors, *b* = 0.18, *SE* = 0.07, *t*(254) = 2.51, *p* = .013, indicating that habit errors increased across blocks (see Fig. 6).

To examine whether the increase in habit errors across blocks was related to the reduction in go RTs, we conducted a participant-level correlation analysis. For each participant, a linear slope across the six experimental blocks was calculated separately for median go RT and square-root-transformed percentage habit errors. More negative go-RT slopes indicated greater speeding across blocks, whereas more positive habit-error slopes indicated greater increases in errors. These slopes were then correlated across participants. There was no significant association between go-RT and habit-error slopes, *r*(49) = − 0.04, *p* = .790, 95% CI [− 0.31, 0.24], indicating that individual differences in the extent of RT speeding across blocks were not associated with individual differences in the increase in habit errors.

### Discussion

The results of experiment 2 are consistent with the prediction that participants would show significant proactive slowing on trials on which they nevertheless made a habit error, but that the level of such proactive slowing on habit error trials would be less than that on correct stop trials. The results are also consistent with the prediction that participants would make more habit errors when the stop step occurred on step 2 of the sequence than when it occurred on step 1 of the sequence. The fact that we did not observe a significant linear increase in habit errors across the sequence, combined with the significant increase in errors from step 1 to step 2, is consistent with the hypothesis that sequential triggering of the default response contributes to errors. A complementary Bayesian analysis supports this interpretation: the posterior for the slope across steps 2–6 was centred near zero, and its upper credible limit corresponded to an increase of only about 3% points between steps 2 and 6. Any further linear increase in errors beyond step 2 is therefore likely to be small. However, this analysis addresses linear change only and does not establish that error rates were equivalent across steps 2–6, so a small increase or a non-linear pattern remains possible. The present data therefore cannot fully rule out a modest contribution from changes in attention across the sequence.

The results are also consistent with the predictions that go trial RTs would decrease, and habit error frequency would increase, across the six blocks of the task. Together, these findings are consistent with the interpretation that performance of the sequence became increasingly automatic over the course of the task, with participants responding faster to the default sequence and finding it increasingly difficult to inhibit the default action when required. However, increasing automaticity is inferred from these behavioural changes rather than directly demonstrated, and alternative explanations such as strategic speeding, fatigue, or changes in motivation cannot be excluded. The lack of a significant correlation between changes in RT and errors across blocks is less straightforward to reconcile with this interpretation but does not necessarily undermine this account. Group-level trends and across-participant correlations address different questions, and the correlation may have been limited by individual differences in strategy, partly dissociable processes underlying speed and inhibition failures, and/or the low reliability of change scores based on few error trials.

## Experiment 3

The results of experiments 1 and 2 both suggest that although proactive control can have substantial beneficial effects on the success of inhibitory control, there are also limitations to such effects. In experiment 1 habit errors continued to occur despite cueing, whilst in experiment 2 habit errors occurred despite significant proactive slowing. To what extent do these results reflect an inherent limitation in the ability to translate proactive control into enhanced performance? Are some errors inevitable even when people attempt to exert some level of proactive control, or does the continued presence errors reflect a lack of motivation? Prior studies have demonstrated that the efficiency of inhibitory control can be enhanced with reward incentives (Leotti & Wager, 2010; Wang et al., 2019; Le et al., 2020), and that such effects may be mediated via effects on proactive control (Verbruggen & McLaren, 2018). Thus, it is possible that providing monetary incentives for successful stopping in the present sequence task might lead to an additional increase in proactive control, over and above that already observed due to cueing, and that this might be accompanied by a further reduction in habit errors.

In the previous study on action switching, Dodds (2025) found that reward incentives led to a significant reduction in action switching errors, but in the absence of any significant effect of reward on proactive slowing. This finding could be interpreted in a number of ways. Firstly, it is possible that effects of reward on cognitive control, at least in the context of the action switching task employed in that study, might be entirely mediated by effects on reactive rather than proactive control. Whilst this interpretation goes somewhat against the grain of previous findings that reward enhances proactive control, it does fit with the observation that reward improved switching performance without exerting any observable effect on proactive slowing. An alternative explanation is that the study was simply underpowered to detect an effect of reward on proactive slowing – indeed, that study was primarily powered to detect the effect of reward on switching success, not proactive slowing, so the lack of significance of the latter effect could plausibly be attributed to a type II error. Finally, it is possible that the lack of effect of reward on proactive control in that study was due to the use of an action switching task and not a pure motor response inhibition task, perhaps, for example, due to the overall difficulty of having to both inhibit the default response and also select a new response at the point of switching.

Therefore, the aim of the final experiment described here was to investigate whether providing reward incentives for successful stopping would lead to (a) an additional increase in proactive slowing on rewarded stop trials over and above that associated with cueing, and (b) an additional decrease in habit errors due to failures of inhibitory control. Participants performed the same sequence stopping task as in experiment 2, with all stop trials cued, but with the variation that in half of the blocks, at the start of a stop trial and alongside the cue, participants saw a picture of a coin that informed them that they could win a small financial bonus on this trial if they stopped successfully. We predicted that reward incentives would lead to significant proactive slowing and a significant reduction in habit errors relative to the no-reward (cue only) condition.

### Method

#### Participants

A pilot study (*N* = 10) was carried out to determine the sample size required to detect significant effects of reward incentives on proactive control and stopping success. Ten participants were recruited from Prolific and completed the reward vs. no-reward version of the sequence stopping task. Paired samples t-tests revealed Cohen’s d effect sizes of 0.74 and 0.51 for the effects of reward on frequency of habit errors and proactive slowing (RTs), respectively, and so the study was powered to detect the smaller effect. A G-power analysis revealed that a sample size of *N* = 33 would be required to detect an effect size of 0.51 with 80% power and an alpha level of 0.05 (2-tailed). For the main experiment, 40 participants (17 females) were recruited from Prolific, with a mean age of 41.9 (SD = 12.4). Thirty-three participants were right-handed. Participants were paid £3.75 for taking part, plus a bonus payment of £0.05 for each correct stop trial in reward blocks (there were 18 such trials so the maximum bonus for each participant was £0.90). The experiment was reviewed and approved by the Exeter Psychology Research Ethics Committee (application ID 6742165). Data were collected in 2025.

#### Task design and procedure

The task design and procedure were exactly the same as experiment 1 except that in three of the blocks participants saw the ‘Stop’ cue on stop trials (no-reward condition), and in the other three blocks (reward condition) participants saw the ‘Stop’ cue and in addition a picture of a 5p coin above. On reward trials, participants received a 5p reward if they responded correctly on every step of the sequence, including the stop step. Participants were informed at the end of each reward block how much extra money they had won on that block, and at the end of the experiment how much money they had won across the whole task. Bonus payments were made through Prolific after the end of the study.

### Results

Two participants’ go step accuracy was < 84% and their data were excluded from all analyses. Mean go step accuracy for the remaining participants was 97% correct (SD = 2.9). As a manipulation check, a one-sample t-test confirmed that habit error rates were significantly greater than zero (M = 6.29%, SD = 6.76%), t(37) = 7.06, *p* < .001 (test conducted on sqrt-transformed values), demonstrating that the task successfully induced habitual responding across the sample.

A paired samples t-test on square root transformed percentage of habit errors revealed significantly fewer errors in the reward condition (M = 4.24%, SD = 5.07) than in the no-reward condition (M = 8.33%, SD = 10.6), t(37) = 2.2, *p* < .05, d = 0.38.

**Fig. 7:**
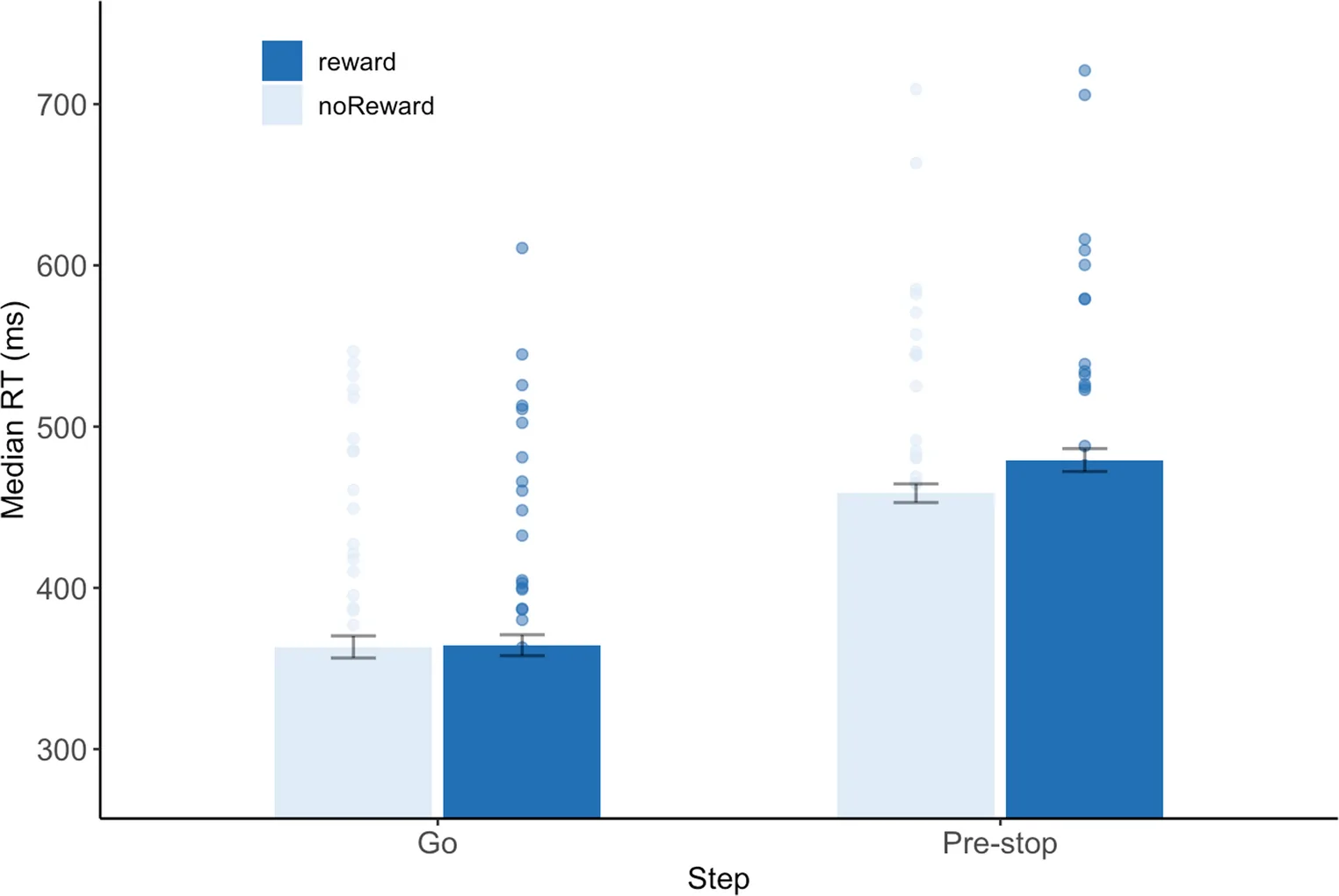
Means of median RTs on go steps and pre-stop steps in the reward and no-reward conditions in experiment 3. Error bars represent SEM; individual points represent participant-level median RTs

To examine effects of reward on proactive slowing, a repeated measures ANOVA was carried out with the factors reward condition (reward, no-reward) and step (go, pre-stop) with median RTs as the dependent variable. This revealed a significant main effect of reward, F(1, 37) = 4.94, *p* = .032, η²_g_ = 0.003, a significant main effect of step, F(1, 37) = 119.84, *p* < .001, η²_g_ = 0.216, and a significant reward x step interaction, F(1, 37) = 6.62, *p* = .014, η²_g_ = 0.002. Follow-up paired samples t-tests revealed that pre-stop step RTs were significantly slower in the reward condition (M = 479 ms, SD = 92) than in the no-reward condition (M = 459 ms, SD = 91), t(37) = 3.5, *p* = .001, d = 0.22, but there was no significant difference in go RTs between the reward condition (M = 364 ms, SD = 112) and the no-reward condition (M = 363 ms, SD = 109), t(37) = 0.16, *p* = .87, d = 0.009 (see Fig. 7).

To examine whether the magnitude of reward-induced proactive slowing was associated with the magnitude of the reduction in habit errors, we conducted a participant-level correlation analysis. For each participant, reward-induced proactive slowing was calculated as the difference in median pre-stop RT between the reward and no-reward conditions, with larger positive values indicating greater reward-induced slowing. Reward-induced reduction in habit errors was calculated as the difference between square-root-transformed percentage errors in the no-reward and reward conditions, with larger positive values indicating a greater reduction in errors following reward. These two effect measures were then correlated across participants. The correlation was positive but did not reach statistical significance, *r*(36) = 0.32, *p* = .053, 95% CI [− 0.003, 0.58].

### Discussion

The results of experiment 3 show that, as predicted, availability of reward incentives for successful stopping led to a significant increase in proactive slowing, which was accompanied by a significant reduction in the frequency of habit errors. We additionally examined whether individual differences in the magnitude of reward-induced proactive slowing were associated with the magnitude of the reduction in habit errors. The correlation was positive, such that participants who showed greater reward-induced slowing tended to show greater reductions in habit errors, although this association did not reach conventional levels of statistical significance. Thus, while the direction of the relationship is consistent with the proposal that increased proactive control contributes to the beneficial effects of reward on inhibitory performance, the correlational analysis provides only tentative evidence for this interpretation.

## General discussion

The present findings have several implications for theories of sequential action control. First, they indicate that the automaticity acquired through sequence learning does not render individual component actions impervious to cognitive control. Participants were able to use advance information to prepare for the inhibition of an upcoming action while continuing to execute the remainder of the learned sequence. Thus, proactive control can be recruited selectively to regulate an individual component action within a learned behavioral sequence. At the same time, the persistence of habit errors despite substantial proactive slowing indicates that the influence of learned sequence representations is not completely overridden by proactive control. Sequential action control can therefore be understood as reflecting an interaction between relatively automatic action triggering and flexible top-down modulation, rather than a simple transition between automatic and controlled modes of behaviour.

These findings are consistent with the notion that limitations in the efficacy of proactive control during action sequence performance may be related to increasing automaticity of the sequence, such that individual actions become increasingly strongly triggered by preceding actions. The concurrent decrease in go-trial RTs and increase in habit errors across blocks provides a behavioural pattern consistent with this interpretation. However, automaticity was not directly measured in the present study, and these changes could potentially also reflect other factors such as strategic speeding, fatigue, or changes in motivation over the course of the experiment. Thus, the evidence for increasing automaticity should be regarded as inferential rather than definitive. This conclusion is also consistent with the pattern of errors in Experiment 2. Habit errors increased from step 1 to step 2 but showed no significant linear increase thereafter, and a Bayesian analysis indicated that any further linear increase in errors was likely to be small. This is consistent with errors being driven by ballistic triggering of the default response by the preceding action, although a small or non-linear contribution from waning attention cannot be ruled out.

The present findings also provide evidence that the extent to which top-down modulation is recruited is itself flexible. Informative cues increased proactive slowing and reduced habit errors, while the addition of reward produced a further increase in proactive slowing and a further reduction in errors. The degree to which an upcoming component action was prepared for inhibition was therefore sensitive to its anticipated behavioural consequences. This suggests that control over learned action sequences is not fixed by the level of automaticity acquired through practice, but can be adjusted according to the anticipated demands and motivational significance of the action.

An important question raised by the effects of reward in Experiment 3 is why participants did not spontaneously engage the maximum possible level of proactive control in the absence of an incentive. One possibility is that proactive control carries costs that make maximal engagement suboptimal when the need for inhibition is relatively infrequent. In the present task, increased proactive control was associated with slower responding on the steps preceding a potential stop, providing a clear temporal cost to increased preparation. More generally, maintaining a goal to inhibit an upcoming response may also impose cognitive or attentional demands, potentially reducing the efficiency with which the sequence can otherwise be performed. From this perspective, participants may allocate proactive control according to the anticipated benefits of doing so, rather than engaging it maximally on every trial. The additional reward in Experiment 3 may have increased the expected value of successful inhibition sufficiently to justify greater investment in proactive control.

Taken together, these findings extend those of previous studies on proactive control and response inhibition by demonstrating that beneficial effects of proactive control on response inhibition can occur when the action to be inhibited is triggered with a relatively high degree of automaticity as part of a highly practised sequence of linked actions. The results also extend those of previous studies in showing that proactive control specifically affects the accuracy of the stopping process. As outlined in the introduction, prior studies that have investigated effects of proactive control on response inhibition have, as far as we are aware, generally used the SST (Doekemeijer et al., 2023; Leunissen et al., 2016; Messel et al., 2019, 2021; Ramautar et al., 2004; Verbruggen & Logan, 2009), in which the primary measure concerns the efficiency of the stopping process, i.e. how fast stopping can occur. The SST uses a tracking procedure to adjust the temporal parameters of the task and maintain overall stopping accuracy at approximately 50% correct, and therefore stopping accuracy is not free to vary across different manipulation conditions. Thus, whilst the SST provides an extremely sensitive measure of stopping efficiency, it is not well suited to addressing questions about the accuracy of stopping. On the other hand, whilst traditional go/no-go tasks *are* designed to measure inhibition success, they will often produce ceiling effects when combined with cueing procedures that indicate the likelihood of an upcoming target. In contrast, the primary focus in the present series of studies concerns the participant’s success in preventing an unwanted action from occurring and the extent to which manipulations of proactive control contribute to this ability. The present paradigm thus allows for an examination of the *magnitude* of any effects of such manipulations on stopping accuracy, which would not be possible in the SST. By allowing stopping accuracy to vary freely whilst avoiding the ceiling effects associated with go/no-go tasks, this approach enables clear conclusions to be drawn about the absolute efficacy of cueing at modulating performance in relation to the possible lower limit of zero errors. Indeed, the results of experiment 3 show that the combination of informative cues and reward incentives reduces the error rate to quite close to zero, demonstrating a clear and substantial impact of these manipulations on the ability to stop a motor response.

The focus on proactive control in the present study is not meant to imply that performance on the sequence stopping task is purely dependent on preparatory processes. As outlined in the introduction, response inhibition involves a combination of proactive and reactive control processes. In the sequence stopping task, informative cues allow participants to slow down in preparation for stopping, but the act of stopping may still require a degree of reactive control. Indeed, one possible explanation for the observed cue- and reward-induced reductions in habit errors is that these manipulations allow proactive slowing to buy time for more efficient reactive control to take place.

The present findings can also be considered in relation to work on trigger failures in response inhibition. In the stop-signal task, successful stopping depends not only on the efficiency of the inhibitory process once initiated, but also on whether the stop process is successfully triggered in the first place (Doekemeijer et al., 2021; Matzke et al., 2017). This distinction may be particularly relevant to the present findings because, although predictive cues produced clear evidence of proactive preparation, participants continued to make some habit errors on cued stop trials. Thus, proactive slowing does not appear to guarantee successful inhibition on every trial. One possibility is that proactive preparation increases the likelihood that inhibitory control is successfully engaged when required, while occasional failures to engage the inhibitory process may nevertheless result in execution of the strongly established motor response. Alternatively, inhibitory control may have been engaged on these trials but may have been insufficiently fast or strong to overcome the ongoing sequential motor response. Our behavioural measures cannot distinguish between these possibilities. The present findings therefore demonstrate that proactive preparation reduces, but does not eliminate, inhibitory control errors, and the trigger-failure framework provides one possible account of why errors may persist despite evidence of advance preparation.

Although we have interpreted the slowing of responses preceding stop trials as reflecting proactive inhibitory control, we acknowledge that this effect may also arise from strategic response caution or expectancy effects. These accounts are not necessarily mutually exclusive, as anticipating a possible stop signal may lead participants to adopt a more cautious response strategy without necessarily involving increased inhibitory preparation at the motor level. Future research could therefore use measures that provide more direct evidence of changes in inhibitory processes. For example, transcranial magnetic stimulation (TMS) could be used to assess changes in motor excitability following stop cues, providing a more direct index of the involvement of motor inhibition. TMS measures could also help distinguish between local and global inhibitory effects by examining whether cue-related changes in excitability are restricted to the motor representation associated with the forthcoming response or extend to other motor representations. Such an approach, informed by work on motor inhibition and excitability (e.g., Duque et al., 2010; Wessel & Aron, 2013) could help determine whether pre-stop slowing reflects enhanced inhibitory preparation per se, rather than response caution or expectancy alone.

## Data Availability

Data were analyzed using R, version 4.4.1 (R Core Team, 2025) and the package ggplot, version 3.5.1 (Wickham, 2016). All data and analysis code are available at https://figshare.com/s/a25a54d4e57672468a7c. We report how we determined the sample size, all data exclusions (if any), all manipulations, and all measures in the study. This study’s design and its analysis were not preregistered.
